# Predicting hypovitaminosis C with LASSO algorithm in adult critically ill patients in surgical intensive care units: a bi-center prospective cohort study

**DOI:** 10.1038/s41598-024-54826-y

**Published:** 2024-03-01

**Authors:** Jie Hu, Jingwen Zhang, Dawei Li, Xin Hu, Qi Li, Wenwen Wang, Jianguo Su, Di Wu, Hongjun Kang, Feihu Zhou

**Affiliations:** 1 Department of Critical Care Medicine, The First Medical Centre, Chinese PLA General Hospital, Beijing, 100853 People’s Republic of China; 2 National Key Laboratory of Kidney Diseases, Beijing, 100853 People’s Republic of China; 3 Department of Critical Care Medicine, The Sixth Medical Centre, Chinese PLA General Hospital, Beijing, 100048 People’s Republic of China; 4 Department of Critical Care Medicine, The Second Affiliated Hospital of Cheeloo Medical College, Shandong University, Jinan, 250013 People’s Republic of China; 5 Department of Critical Care Medicine, NingXia Chinese Medicine Research Center, Yinchuan, 750021 People’s Republic of China; 6 Key Laboratory of Biomechanics and Mechanobiology, Ministry of Education, Beijing Advanced Innovation Center for Biomedical Engineering, School of Biological Science and Medical Engineering, Beihang University, Beijing, 100083 People’s Republic of China; 7 Medical Engineering Laboratory of Chinese, PLA General Hospital, Beijing, 100853 People’s Republic of China

**Keywords:** Diseases, Health care, Risk factors

## Abstract

Vitamin C played pleiotropic roles in critical illness and vitamin C insufficiency was predictive of the development of multiple organ failure. Currently, the prevalence of vitamin C insufficiency in Chinese critically ill patients is rarely determined and there are no established bedside tools to predict hypovitaminosis C. To develop a nomogram to identify patients with high risk of hypovitaminosis C, we performed a bi-center prospective cohort study at two ICUs of the first and sixth medical center in PLA General Hospital, Beijing, China from May 6th to July 31st, 2021 We identified 322 eligible patients. 62.4% patients were hypovitaminosis C. 7 features, including source of infection, the level of serum albumin, age, male gender, sepsis, vascular disease, and wasting of vitamin C by the kidney, were selected using LASSO algorithm and therefore included in the nomogram. In the testing set, our model showed moderate discrimination ability with areas under the curve of 0.75 [0.64–0.84]. Variable importance evaluated by SHAP value highlighted two novel important predictors, i.e., abdominal infection and the level of serum albumin. In conclusion, we first reported a high burden of vitamin C insufficiency in Chinese adult patient in the ICU. We also constructed a prediction model to timely identify patients with high risk of hypovitaminosis C, which allows the clinicians to choose appropriate candidates for Vitamin C repletion in clinical practice or clinical trials.

## Introduction

Vitamin C is an essential nutrient which cannot be synthesized or stored in humans^1^. It played pleiotropic roles in critical illness in terms of anti-oxidative injury, anti-inflammation, and synthesis of endogenous catecholamine, etc.^1^. Vitamin C insufficiency [including hypovitaminosis C (i.e., plasma vitamin C < 23 μmol/L) and vitamin C deficiency (i.e., plasma vitamin C < 11 μmol/L)] is commonly seen in critically ill patients, and measurements seem to be much lower in patients with sepsis^2^.

Low plasma concentration of vitamin C was predictive of the development of multiple organ failure^3^. Given scientific rationale of vitamin C treatment in sepsis, interest in supplementation of vitamin C has rapidly increased. However, from the inception of the “cocktail therapy (i.e., hydrocortisone, ascorbic acid [vitamin C], and thiamine, HAT)” strategy generated in a cohort study^4^, most of the subsequent randomized clinical trials (RCTs), including ACTS^5^, ORANGES^6^, HYVCTTSSS^7^, VITAMINS^8^, and VICTAS^9^, failed to meet the pre-defined primary endpoints, such as 28d or 30d mortality, or length of ICU stay, etc. Moreover, vitamin C using as monotherapy in sepsis also confirmed the aformentioned findings. In CITRIS-ALI study conducted among patients with sepsis and ARDS, vitamin C did not significantly improve organ dysfunction scores or alter markers of inflammation and vascular injury^10^. LOVIT study^11^ and its secondary analysis^12^ again proved invalidity of vitamin C in sepsis. Why effect of vitamin C on sepsis or septic shock among studies was so conflict? As an adjunctive therapy, it is reasonable to assume that only patients with vitamin C insufficiency could benefit from intravenous administration. However, none of the aforementioned RCTs have listed vitamin C insufficiency as the inclusion criteria since timely measurement of plasma vitamin C before randomization is not available. Hence, bedside screening for patients with high risk of vitamin C insufficiency is warranted.

In our prospective cohort study, we sought to describe the prevalence of vitamin C insufficiency in Chinese adult patients in the intensive care units (ICU), and establish a nomogram for clinicians to identify patients with high risk of hypovitaminosis C at ICU admission.

## Methods

### Study design and general information

The study was a bi-center, prospective cohort study conducted at two ICUs of the first and sixth medical center in PLA General Hospital, Beijing, China from May 6th to July 31st, 2021 (Registered on http://www.chictr.org.cn, ChiCTR2100043451 on 3rd June, 2021). Approval for the study was granted by the Ethics Committee of PLA General Hospital. Written informed consent forms were obtained by the patients or their legal proxies when patient consent was not available due to critical illness within 24 h after admission. These patients were followed up until death, discharge from the ICUs, or till 7 days, whichever applied to the patient. All methods were performed in accordance with the Declaration of Helsinki. In performing this study, we followed the recommendations established in the Transparency Reporting of a multivariable prediction model for Individual Prognosis or Diagnosis (TRIPOD) initiative^13^.

### Inclusion criteria

All patients over 18 years old admitted to the ICUs of the first and sixth medical center in PLA general hospital.

### Exclusion criteria

Being pregnant or postpartum;
Prior administration of intravenous Vitamin C within 1 week;
3.Post-cardiopulmonary resuscitation;
Discharged or having incomplete data within 24 h of admission;
Consent forms cannot be obtained.

If patients were admitted to the ICUs for multiple times, only data from the first admission was recorded during the study period.

### Outcome measures

The primary outcome was hypovitaminosis C, and the secondary outcome was vitamin C deficiency^2^. Blood samples were harvested immediately after the written consent forms were signed. Plasma Vitamin C were measured via Qlife Lab 9000 HPLC system (Qlife, Nanjing, China) for research purpose^14^.

### Predictors

A list of candidate predictors with a known association with hypovitaminosis C were determined a priori, including age^15^, gender, obesity^16^, smoking^17^, and infectious disease^18^. In addition, comorbidities such as chronic respiratory diseases^19^, diabetes^20^, hypertension^21^, moderate to severe heart failure^22^, cancer with chemotherapy^23^, disorders of the gastrointestinal tract^24^ and liver cirrhosis^25^, and factors which lead to wasting of vitamin C by the kidneys^24^ were also included. Since vitamin C is actively accumulated in human granulocytes^26^ and vitamin C might be depleted in acute phase response due to oxidative stress, we assumed that biomarkers such as neutrophil lymphocyte ratio (NLR), C-reactive protein (CRP), and procalcitonin (PCT) might also be promising predictors. As vascular disease was associated with low vitamin C intake and high plasma fibrinogen concentration^27^, primary diagnosis of vascular disease and biomarkers including fibrinogen, D-dimer and prothrombin time (PT) were also collected as eligible predictors. In addition, serum lactate level was included for proper diagnosis of septic shock^28^. Sepsis and septic shock were established according to Sepsis 3.0 definition^28^ by a senior clinician unaware of the research purpose.

In general, our study encompasses demographics, Sequential organ function assessment (SOFA) score, comorbidities and presumed risk factors of vitamin C insufficiency, laboratory tests (determined by the primary investigators), and source of infection (if applicable). The clinical events during 7 days follow-up were also documented only to fully describe characteristics of enrolled patients. The details of included variables were presented in Supplemental Table S1.

### Sample size calculation

The sample size was calculated to ensure that there were enough records for the development of the model. In terms of binary outcome, events per variable of 5 is needed^29^. With a presumed prevalence of hypovitaminosis C around 60% in adult patients^17,30,31^ in the ICU and 27 variables as potential risk factors, at least (27 × 5/0.6) = 225 samples are needed for the training dataset.

### Statistical analysis

A statistical analysis protocol was prepared prior to data collection. Only participants with available measurements of plasma vitamin C were included in the final analysis. With regard to missing data, the “missing at random” assumption was applied. Missing data of continuous variables other than levels of plasma vitamin C were imputed with the median value of the entire feature column, and variables with a missing rate more than 5% were excluded.

The included patients were randomly divided into a training set and a testing set at a ratio of 7:3. For comparison of variables between groups, continuous data were presented as median with interquartile range (IQR, 25–75th percentiles) and compared with Mann–Whitney U test, while categorical variables were presented as frequencies and compared with a chi-square test. All analyses were two-sided, and a *p* value of less than 0.05 was considered statistically significant.

Prediction models were developed using logistic regression. Univariable models were fit for each predictor to assess individual associations. Restricted cubic spline (RCS) with three knots was used to account for potential non-linearity in the association between continuous predictors and the outcome. When evidence of a non-linear relationship was found, RCS was further used to model the effects of those predictors in multivariable model.

In the training set, variable selection was performed using a least absolute shrinkage and selection operator (LASSO) approach, where data values are shrunk towards a central point as the mean, i.e., forcing the β-coefficients of each factor to be zero^32^. All the listed eligible variables assessed in the univariable analyses were included in the LASSO algorithm. The predictors selected by LASSO algorithm were finally included in a logistic regression model.

Model performance was assessed as follows. The area under the receiver-operating-characteristics curve (AUROC) was deployed to evaluate the model discrimination. Bootstrapping based methods with 1000 resamples were applied to adjust for the overfitting^33^. Resampling model calibration through calibration curve was conducted to evaluate the model calibration. F1 score^34^, accuracy ^35^, sensitivity, specificity, positive predictive value (PPV), and negative predictive value (NPV) were also calculated. Decision curve analysis (DCA) was implemented to assess the applicability of the prediction model^36^. We reported the above measurements of model performance both in training and testing dataset.

For better interpretation of the prediction model, a nomogram was established based on a logistic regression with variables selected by LASSO algorithm. The Shapley additive explanation (SHAP) was also applied to test how individual factors in this analysis contributed to hypovitaminosis C.

We also developed a prediction model for vitamin C deficiency. To meet the thumb rule of events per variable^29^, the model was derived from the complete cohort and the performances was adjusted with 1,000-fold bootstrapping.

Statistical analyses were conducted using R version 3.5.3 (R Core Team, R Foundation for Statistical Computing, Vienna, Austria) with relevant packages (tableone, rms, ROCR, glmnet, pROC, plyr, Hmisc, magrittr, rpart). Data pre-processing, model development and validation, and visualization of results are implemented using Python version 3.9 with related packages (pandas, numpy, scikit-learn, matplotlib).

## Results

### Patient characteristics

A total of 322 patients with available measurements of plasma vitamin C were eligible for the present study, who were divided into a training dataset with 225 patients and a testing dataset with 97 patients. Supplemental Fig. S1 shows the flowchart of the study. Overall, the median age was 68.00 [58.00, 79.00] years old, and 64.9% were male. 75.5% patients received mechanical ventilation. 13.7% developed acute kidney injury and 8.1% received renal replacement therapy within 7 days. 4% died within 7 follow-up days. 62.4% patients were hypovitaminosis C and 34.2% patients were vitamin C deficiency. Among all the candidate predictors, only CRP had missing values and therefore was imputed with the median of overall population.

Table 1 showed twenty-seven candidate variables differentiating between the training and testing sets, as well as the clinical events. The only significant difference found in the validation set was a higher serum level of D-dimer, which was negligible.

**Table 1:** Comparison of patient characteristics between the derivation and validation groups.

Variable	Overall (n = 322)	Training (n = 225)	Testing (n = 97)	*p*
Demographics				
Age, years, median [IQR]	68.00 [58.00, 79.00]	67.00 [57.00, 78.00]	68.00 [59.00, 80.00]	0.33
Male gender, n (%)	209 (64.9)	149 (66.2)	60 (61.9)	0.53
Body mass index, n (%)				0.87
18.5 ≤ BMI < 25	157 (48.8)	113 (50.2)	44 (45.4)	
BMI < 18.5	33 (10.2)	22 (9.8)	11 (11.3)	
25 ≤ BMI < 30	111 (34.5)	76 (33.8)	35 (36.1)	
BMI ≥ 30	21 (6.5)	14 (6.2)	7 (7.2)	
Smoker, n (%)	122 (37.9)	90 (40.0)	32 (33.0)	0.29
Comorbidities				
Moderate to severe heart failure, n (%)	22 (6.8)	15 (6.7)	7 (7.2)	1.00
Hypertension, n (%)	142 (44.1)	103 (45.8)	39 (40.2)	0.42
Diabetes, n (%)	71 (22.0)	46 (20.4)	25 (25.8)	0.36
Pulmonary disease, n (%)	29 (9.0)	22 (9.8)	7 (7.2)	0.60
Wasting of vitamin C by the kidney, n (%)	19 (5.9)	13 (5.8)	6 (6.2)	1.00
Digestive system disorder, n (%)	28 (8.7)	22 (9.8)	6 (6.2)	0.40
Cancer with chemotherapy, n (%)	23 (7.1)	16 (7.1)	7 (7.2)	1.00
Primary diagnosis at admission to the ICU				
SOFA score, median [IQR]	3.00 [2.00, 6.00]	3.00 [2.00, 6.00]	4.00 [2.00, 6.00]	0.34
Sepsis, n (%)				0.73
Sepsis, n (%)	136 (42.2)	93 (41.3)	43 (44.3)	
Septic shock, n (%)	36 (11.2)	24 (10.7)	12 (12.4)	
Source of infection, n (%)				0.60
Abdominal, n (%)	80 (24.8)	53 (23.6)	27 (27.8)	
Others, n (%)	56 (17.4)	38 (16.9)	18 (18.6)	
Abdominal surgery, n (%)	126 (39.1)	89 (39.6)	37 (38.1)	0.91
Vascular disease, %	27 (8.4)	18 (8.0)	9 (9.3)	0.87
Lab tests at admission to the ICU				
NLR, median [IQR]	11.65 [6.92, 17.70]	11.81 [6.86, 17.69]	11.10 [7.06, 17.70]	0.75
CRP, mg/dL, median [IQR]	2.66 [1.00, 7.14]	2.66 [1.00, 7.17]	2.82 [0.92, 7.07]	0.77
PCT, ng/ml, median [IQR]	0.25 [0.07, 1.29]	0.25 [0.07, 1.23]	0.25 [0.07, 1.49]	0.86
Fib, g/L, median [IQR]	3.48 [2.76, 4.32]	3.47 [2.76, 4.27]	3.52 [2.76, 4.43]	0.84
PT, s, median [IQR]	14.50 [13.30, 15.78]	14.40 [13.20, 15.70]	14.90 [13.60, 15.90]	0.07
DD, μg/ml, median [IQR]	2.72 [1.29, 5.19]	2.64 [1.21, 4.58]	2.99 [1.65, 6.26]	0.04
LDH, U/L, median [IQR]	201.20 [156.25, 282.08]	201.20 [159.70, 276.20]	201.20 [154.40, 286.80]	0.93
Alb, g/L, median [IQR]	32.40 [29.20, 34.98]	32.50 [29.30, 35.50]	32.10 [28.70, 34.00]	0.14
Cr, umol/L, median [IQR]	75.45 [59.70, 103.40]	75.30 [59.70, 99.30]	76.90 [59.70, 106.10]	0.65
NT-pro BNP, pg/ml, median [IQR]	254.90 [85.50, 754.80]	254.90 [83.20, 750.00]	277.60 [93.00, 775.10]	0.63
Lac, mmol/L, median [IQR]	1.45 [1.00, 2.40]	1.50 [1.00, 2.50]	1.40 [1.00, 2.20]	0.23
Clinical events				
Mechanical ventilation, n (%)	243 (75.5)	172 (76.4)	71 (73.2)	0.63
AKI, n (%)	44 (13.7)	31 (13.8)	13 (13.4)	1.00
RRT, n (%)	26 (8.1)	21 (9.3)	5 (5.2)	0.30
Death, n (%)	13 (4.0)	10 (4.4)	3 (3.1)	0.80
Outcome				
Plasma levels of Vitamin C, μmol/L, median [IQR]	17.01 [8.48, 34.33]	16.93 [8.29, 34.93]	19.19 [8.94, 30.39]	0.82
Hypovitaminosis C, n (%)	201 (62.4)	140 (62.2)	61 (62.9)	1.00
Vitamin C deficiency, n (%)	110 (34.2)	76 (33.8)	34 (35.1)	0.98

*NLR* neutrophil lymphocyte ratio, *CRP* C-reactive protein, *PCT* procalcitonin, *Fib* fibrinogen, *DD* D-dimer, *PT* prothrombin time, *LDH* lactic dehydrogenase, *Alb* albumin, *Cr* creatinine, *NT-pro BNP* NT-pro brain natriuretic peptide, *AKI* acute kidney injury, *RRT* renal replacement therapy.

Comparison of patient characteristic grouped by hypovitaminosis C or vitamin C deficiency was demonstrated in Supplemental Tables S2 and S3. Patients with hypovitaminosis C or vitamin C deficiency were more likely to have older age, higher prevalence of vascular diseases, sepsis or septic shock, abdominal sepsis, higher SOFA score, higher levels of CRP, PCT, D-dimer, NT-pro brain natriuretic peptide (NT-pro BNP), creatinine, and prolonged PT but lower levels of albumin. Moreover, substantial differences in 7 days mortality and prolonged duration of mechanical ventilation were observed between the two groups. Additionally, patients with vitamin C deficiency were more likely to have lower BMI.

### Development of a prediction model in training set

In the training set, 144 (62.2%) patients were hypovitaminosis C. In the univariable logistic regression analyses, age, levels of albumin, CRP, PT, NT-pro BNP, D-dimer, PCT, sepsis, SOFA score, source of infection, and vascular disease were found to be associated with hypovitaminosis C. The ORs with 95% CI are shown in Table 2. It is noted that NT-pro BNP, CRP, D-dimer, PCT, PT, SOFA score showed non-linear relationship with hypovitaminosis C (data not shown).

**Table 2:** Univariable and multivariable logistic regression for hypovitaminosis C.

Characteristics	Univariable analysis			Multivariable analysis		
	OR	95%CI	P value	OR	95%CI	*p* value
Age	1.02	1.00–1.03	0.01	1.02	1.01–1.04	0.01
Body mass index						
BMI < 18.5	0.85	0.34–2.13	0.72			
25 ≤ BMI < 30	0.97	0.59–1.59	0.90			
BMI ≥ 30	2.36	0.97–5.77	0.06			
Male gender	1.38	0.86–2.20	0.18	1.42	0.82–2.45	0.21
Smoker	1.05	0.66–1.67	0.84			
Moderate to severe heart failure	1.66	0.63–4.36	0.31			
Hypertension	0.97	0.61–1.52	0.88			
Diabetes	1.34	0.77–2.34	0.31			
Pulmonary disease	0.84	0.39–1.82	0.66			
Wasting of Vitamin C by the kidney	2.36	0.76–7.28	0.14	2.70	0.79–9.15	0.11
Digestive system disorder	0.67	0.31–1.46	0.31			
Cancer with chemotherapy	1.41	0.56–3.53	0.46			
Vascular disease	8.45	1.96–36.35	< 0.001	9.28	2.04–42.20	0.004
Abdominal surgery	1.28	0.80–2.03	0.31			
Sepsis	3.21	1.95–5.30	< 0.001	1.87	0.91–3.86	0.09
Septic shock	4.61	1.90–11.17	< 0.001	2.33	0.79–6.86	0.12
Abdominal infection	2.81	1.44–5.49	< 0.001	2.31	1.02–5.22	0.04
Others source of infection	3.47	1.89–6.38	< 0.001	1.77	0.73–4.30	0.21
SOFA score	1.12	1.04–1.21	< 0.001			
NLR	1.01	0.99–1.02	0.36			
CRP	1.09	1.03–1.15	< 0.001			
PCT	1.01	0.99–1.03	0.26			
PT	1.12	1.03–1.22	0.01			
Fib	1.00	0.85–1.18	1			
DD	1.03	0.98–1.09	0.24			
LDH	1.00	1.00–1.00	0.57			
Alb	0.86	0.82–0.91	< 0.001	0.89	0.84–0.94	0.000
Cr	1.00	1–1.01	0.05			
NT-pro BNP	1.00	1–1.0001	0.32			
Lac	1.03	0.92–1.15	0.59			

All 27 variables were included in the LASSO algorithm to perform feature selection to find a balance between model simplicity and accuracy in the training set. When the adjustment parameter was lambda.min (λ = 0.033), 7 features with nonzero coefficients were selected as important indices for the prediction of hypovitaminosis C (Supplemental Fig. S2), including source of infection, sepsis, serum albumin, age,male gender, wasting vitamin C by kidney, and vascular disease. The 7 features were included in the final logistic regression model. Notably, age (OR 1.02; 95% CI 1.01–1.04; *p* = 0.010), vascular disease (OR 9.28; 95% CI 2.04–42.20; *p* = 0.004), infection originated from abdomen (OR 2.31; 95% CI 1.02–5.22; *p* = 0.044), and level of albumin (OR 0.89; 95% CI 0.84–0.94; *p* = 0.000) were shown to be associated with hypovitaminosis C (Table 2).

**Figure 1:**
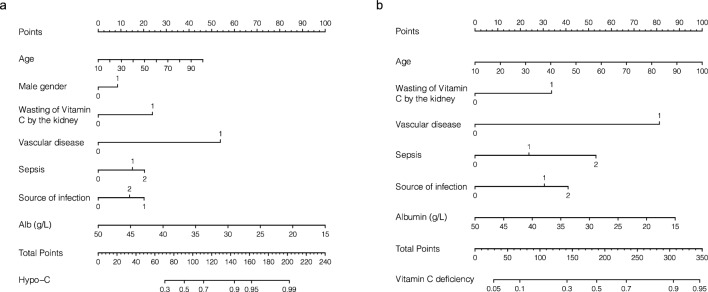
The nomogram for hypovitaminosis C (**a**) and Vitamin C deficiency (**b**).

To better interpretate the prediction, a nomogram was constructed, as described in Fig. 1a. Each predictor was corresponded to a single score shown on the top line of the nomogram. The total score of each patient is the summation of each single score. On the bottom line of the nomogram, the probabilities of hypovitaminosis C in ICU patients were predicted in terms of total score.

**Figure 2:**
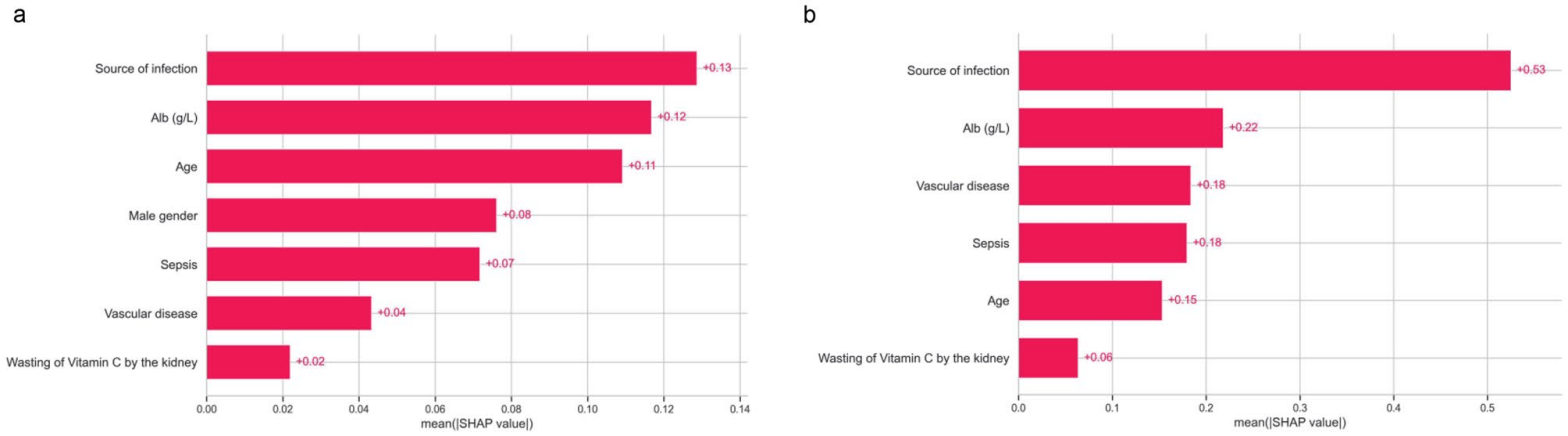
Contributions of input features to prediction. This importance matrix plot depicts the importance of each feature in the development of the final predictive model. The higher the SHAP value of a feature, the higher the probability of hypovitaminosis C (**a**) or vitamin C deficiency (**b**).

To evaluate the contribution of any particular feature to the difference between actual and mean predictions, the SHAP values of 7 selected features in the predictive model were reported in Fig. 2a. It was noted that source of infection and levels of serum albumin were shown to be the most important predictors of hypovitaminosis C. Patients who had infection originated from abdomen were more likely to have hypovitaminosis C. Moreover, lower albumin level was a significant factor in the model against hypovitaminosis C. We further developed a WeChat based mini program named “HYPOVC” for bedside application (Supplemental Fig. S4).

Given the thumb rule of events per variable^29^, prediction model against Vitamin C deficiency was developed in the complete cohort. The ORs with 95% CI in the univariable and multivariable analyses were shown in Supplemental Table S4. All 27 variables were included in the LASSO algorithm to perform feature selection. When the adjustment parameter was lambda.min (λ = 0.034), 6 predictors were selected (Supplemental Fig. S5) and therefore included in the final model, which were source of infection, sepsis, serum albumin, age, wasting vitamin C by kidney, and vascular disease . Age (OR 1.02; 95% CI 1.00–1.04; *p* = 0.016), vascular disease (OR 4.50; 95% CI 1.84–11.02; *p* = 0.001), and septic shock (OR 2.68; 95% CI 1.01–7.15; *p* = 0.048) were shown to be independently associated with vitamin C deficiency. The nomogram of vitamin C deficiency and SHAP values were presented in Figs. 1b, and 2b, respectively.

### Evaluation and validation of prediction model

Performances of the prediction model against hypovitaminosis C were presented in Table 3. The bootstrap-adjusted C index was 0.76 [0.69–0.83] and 0.75 [0.64–0.84] in the training and testing set, respectively. The ROC curve was shown in Supplemental Fig. S3a and b. In Supplemental Fig. S3c and d, the calibration curves indicated that there was good consistency between the predicted values and the actual observed values. As shown in Supplemental Fig. S3e and f, DCA demonstrated that the nomogram had good overall net benefits.

**Table 3:** Performance of the prediction model against Hypovitaminosis in the training and testing sets.

	AUC [CI]	Accuracy	Sensitivity	Specificity	F1	PPV	NPV
Training set							
Lasso with LR	0.76 [0.69–0.83]	0.66 [0.60–0.72]	0.65 [0.57–0.72]	0.68 [0.59–0.78]	0.70 [0.64–0.76]	0.77 [0.70–0.84]	0.54 [0.44–0.63]
Testing set							
Lasso with LR	0.75[0.64–0.84]	0.67[0.58–0.76]	0.71[0.60–0.82]	0.61 [0.46–0.78]	0.73 [0.64–0.81]	0.75 [0.63–0.86]	0.56 [0.41–0.71]

*NPV* negative predictive value, *PPV* positive predictive value.

The aforementioned model performances were also evaluated in prediction model against vitamin C deficiency and adjusted with 1000-fold bootstrapping, as shown in Supplemental Table S5 and Supplemental Fig. S6. The bootstrap-adjusted C index was 0.72 [0.66–0.77].

## Discussion

In this study of 322 Chinese adult patients in the ICU, nearly two thirds of them were hypovitaminosis C and one third of them were vitamin C deficiency. To our knowledge, this is the first study that constructed a bedside tool to identify patients with high risk of hypovitaminosis C with data available at admission to the ICU, which allows clinicians to screen eligible patients who might be benefit from vitamin C supplementation. Our study also highlighted infection originated from abdomen and serum albumin as novel predictors of hypovitaminosis C.

The epidemiology of vitamin C insufficiency was fully determined in lots of countries other than China. Our study reported a high burden of vitamin C insufficiency in Chinese ICU patients, which was in consistent with data from a cohort of 44 critical ill patients in New Zealand^2^. It is worth mentioning that abdominal surgeries were performed in 39.1% of patients in our cohort, indicating antecedent malabsorption of vitamin C. Since vitamin C cannot be synthesized by humans but only absorbed via enteral or parenteral nutrition^1^, the prevalence of vitamin C insufficiency still needs to be further determined in medical ICUs.

Aside from advanced age^15^, male gender^16^, sepsis or septic shock^18^, vascular disease^27^, and factors which leads to wasting of vitamin C by the kidneys^24^, our study also identified two novel important predictors of hypovitaminosis C, i.e. abdominal sepsis and serum albumin, using variable importance analysis based on SHAP value (the change in the prediction when the feature is added compared with the baseline for a given prediction^37^). Infection originated from abdomen implied malabsorption of vitamin C, and thereby was related with vitamin C insufficiency. Moreover, hypoalbuminemia was considered as a severity marker of underlying pulmonary capillary leakage in patients with COVID-19^38^, while administration of vitamin C could protect against vascular leakage in murine abdominal sepsis^39^, indicating underlying association between hypoalbuminemia and vitamin C insufficiency.

From the inception of the index cohort study^4^, in which administration of HAT remarkably reduced mortality of sepsis, the effect of vitamin C on sepsis or septic shock has generated deeply debate since plenty of subsequent RCTs using either HAT therapy^5,6,8,9^, or monotherapy of vitamin C^11,40^ failed to verify the improvement of survival. The common perspective, as a recent meta-analysis indicated, is that vitamin C might be able to improve delta SOFA score and reduce the duration of vasopressor use, but is not associated with reduction in short-term mortality^41^. In our opinion, the aforementioned controversy could be prominently attributed to discrepant timing^42^, duration^43,44^, dose^45^ of vitamin C administration, and more importantly, the inclusion criteria during patient recruitment.

Candidates in the current clinical trials were consist of plenty patients with pulmonary infection^5,6,8,9,11,40^. However, a recent animal study demonstrated that vitamin C therapy only worked in intra-abdominal sepsis other than pneumonia due to different inflammatory responses to infection^34^, indicating that pulmonary sepsis should be excluded in the future study. Therefore, in CITRIS-ALI study^10^ conducted only in patients with sepsis and ARDS, a 96-h infusion of vitamin C did not significantly improve organ dysfunction or vascular injury. It was further confirmed by our study since only intra-abdominal infection was associated with hypovitaminosis C rather than other resources. More importantly, patients in those trials^5,6,8,9,11,40^ were not all vitamin C insufficiency, and therefore would not benefit from vitamin C administration. Only two studies measured the baseline levels of vitamin C after patient recruitment. In ORANGES^6^, half of the enrolled patients were hypovitaminosis C, and the average levels of plasma vitamin C was higher in the treatment group (29.6 ± 56.8 μmol/L vs. 27.3 ± 22.7 μmol/L), albeit no statistical difference. The same story repeated in LOVIT study (20.6 ± 70.6 vs. 19.1 ± 39.7 μmol/L)^11^. It is noted that levels of plasma vitamin C were 14.1 ± 11.8 μmol/L in the treatment group in the index study^4^. Our study further elucidated that critical ill patient with hypovitaminosis C had higher 7 days mortality and prolonged duration of mechanical ventilation. Therefore, we speculated that the efficacy of vitamin C supplementation can only be observed among patients with vitamin C insufficiency, and that’s why identification of those patients before randomization is warranted.

Measurement of plasma vitamin C levels involves cumbersome techniques (direct measurement by HPLC or indirect measurement by RedoxSYS System^46^) that are impossibly to be available in all hospital laboratories and unlikely to provide a rapid turnaround time. Our study makes it possible to identify patients with high risk of hypovitaminosis C at admission to the ICU, and thus chose appropriate candidates for clinical trials before randomization. For better understanding and general application in the clinical settings, we chose LASSO algorithm with logistic regression in the model development process and further described it in a nomogram. The included predictors were all available at admission. Our Model achieved moderate discrimination and calibration both in the training and testing sets. However, given the small sample size of our cohort, we were not able to validate the model against vitamin C deficiency. In general, we believe that this study developed a promising predictive model of hypovitaminosis C at ICU admission.

Our study has several limitations. First, the enrolled candidates came from two ICUs in which most of the patients were originated from department of general surgery. The prevalence of vitamin C insufficiency might be overestimated due to malabsorption. Therefore, external validation to determine generalizability of our model is warranted, especially among patients from medical ICU. Second, the number of events per variable of vitamin C deficiency is relatively small. The performance of the model was only adjusted with bootstrapping-based method in the derivation cohort per se, and thus overfitting could not be overlooked.

## Conclusions

In our prospective cohort study, we reported a high burden of hypovitaminosis C and vitamin C deficiency in Chinese adult critical ill patients. We for the first-time developed a predictive model and constructed a nomogram to predict the risk of hypovitaminosis C using variables that are commonly available at ICU admission.

### Supplementary Information

Supplementary Information.

## Data Availability

The data are available for other investigators’ use under reasonable request. Please contact the corresponding author for access requests.
