# Supplementary material for: Predicting hypovitaminosis C with LASSO algorithm in adult critically ill patients in surgical intensive care units: a bi-center prospective cohort study
Source: Sci Rep. 2024 Mar 1;14:5073. doi: 10.1038/s41598-024-54826-y (PMC10907613; doi:10.1038/s41598-024-54826-y)
Supplement: Supplementary file 1 — Supplementary Information. [file 41598_2024_54826_MOESM1_ESM.docx]

**Supplementary materials**

**Predicting Hypovitaminosis C with LASSO algorithm in Adult Critically Ill Patients in Surgical Intensive Care Units: a Bi-center Prospective Cohort Study**

Jie Hu^1,2#^, Jingwen Zhang^1#^, Dawei Li^3^, Xin Hu^1^, Qi Li^3^, Wenwen Wang^4^, Jianguo Su^5^, Di Wu^6^, Hongjun Kang^1^, Feihu Zhou^1,7*^

^1^ Department of Critical Care Medicine, The First Medical Centre, Chinese PLA General Hospital, Beijing, 100853, People's Republic of China.
^2^ State Key Laboratory of Kidney Diseases, National Clinical Research Center for Kidney Diseases, Beijing, 100853, People's Republic of China.
^3^ Department of Critical Care Medicine, The Sixth Medical Centre, Chinese PLA General Hospital, Beijing, 100048, People's Republic of China.
^4^ Department of Emergency, The Second Hospital of Shandong University, Jinan, 250013, People's Republic of China.

^5^Department of Critical Care Medicine, NingXia Chinese medicine Research Center，YinChuan,750021,People's Republic of China.

^6^ Key Laboratory of Biomechanics and Mechanobiology, Ministry of Education, Beijing Advanced Innovation Center for Biomedical Engineering，School of Biological Science and Medical Engineering, Beihang University, Beijing, 100083, People's Republic of China.

^7^ Medical Engineering Laboratory of Chinese PLA General Hospital, Beijing, 100853, People's Republic of China.

**Table S1** Details of eligible predictors

**Fig. S1** Flowchart of the study

**Table S2** Comparison of patient characteristics between the patients with or without hypovitaminosis C

**Table S3** Comparison of patient characteristics between the patients with or without Vitamin C deficiency

**Fig. S2** The prediction model for hypovitaminosis C established by LASSO regression with five fold cross-validation.

**Fig. S3** Evaluation and validation of prediction model for Vitamin C Deficiency. The ROC curve, Calibration curve, Decision curve analyses for nomogram in the training set(a, c e) and testing set (b, d, f).

**Fig. S4** Screenshot of WeChat App.

**Table S4** Odds ratio for Vitamin C deficiency in univariable and multivariable analyses

**Fig. S5** The prediction model for vitamin C deficiency established by LASSO regression with five fold cross-validation.

**Table S5** Predictive performance of the model generated to Vitamin C deficiency

**Fig. S6** The performance of prediction model for vitamin C deficiency.

**Table S1 Details of eligible predictors**

| Variables | Definition |
| --- | --- |
| *Risk factors* |  |
| *Demographics* |  |
| Age, yrs |  |
| Body mass index, kg/m^2^ | 1: 18.5≤BMI<25, 2: BMI<18.5, 3:25< BMI≤30, 4: BMI >30 |
| Gender | 1: male; 0: female |
| Smoker | 1: current smoker; 0: previous smoker or never smoke |
| *Comorbidities* |  |
| Moderate to severe heart failure | 1: NYHA III/IV; 0: NYHA I/II |
| Hypertension | 1: history of hypertension, on anti-hypertension medication, or blood pressure over 140/90 mmHg at admission for at least two times, 0: none of the situations above |
| Pulmonary disease | 1: diagnosis of chronic obstructive pulmonary disease or asthma; 0: none of the situations above |
| Wasting of Vitamin C by the kidney | 1: on hemodialysis or peritoneal dialysis before ICU admission, or use of diuretics such as loop diuretics or spirolactone, or diagnosis of chronic kidney disease; 0: none of the situations above |
| Digestive system disorder | 1: diagnosis of cirrhosis, or inflammatory bowel disease, or Crohn’s disease, or history of gastrointestinal surgery; 0: none of the situations above |
| Diabetes | 1: diagnosis of diabetes and on insulin or antidiabetic medication, or fasting glucose level above 126 mg/dL (7 mmol/L) at admission, 0: none of the situations above |
| Cancer with chemotherapy, n (%) | 1: diagnosis of cancer requiring chemotherapy in the last 5 years or other targeting therapy; 0: none of the situations above |
| *Diagnosis* |  |
| SOFA score | Sequential organ failure assessment score |
| Abdominal surgery | 1: admitted to the ICU undergoing abdominal surgery; 0: admitted to the ICU without abdominal surgery |
| Sepsis | 1: with suspected infectious resource and SOFA≥2; 2: a subset of sepsis with persisting hypotension requiring vasopressors to maintain MAP≥65 mm Hg and having a serum lactate level >2 mmol/L (18 mg/dL) despite adequate volume resuscitation; 0: none of the situation above |
| Source of infection | Infection originated from 1: intra-abdominal (including liver and biliary tract); 2: others; 0: none of the situations above |
| Vascular disease | 1: admitted to the ICU due to aortic aneurysm, aortic dissection, peripheral vascular disease and recent stroke or myocardial infarction; 0: none of the situation above |
| *Labs* |  |
| NLR | Percentage of neutrophils divided by percentage of lymphocytes at admission to the ICU |
| CRP | Levels of c-reactive protein at admission to the ICU |
| PCT | Levels of procalcitonin at admission to the ICU |
| Fib | Levels of fibrinogen at admission to the ICU |
| DD | Levels of D-dimer at admission to the ICU |
| PT | Levels of prothrombin time at admission to the ICU |
| LDH | Levels of [lactic](javascript:;) [dehydrogenase](javascript:;) at admission to the ICU |
| Alb | Levels of albumin at admission to the ICU |
| Cr | Levels of creatinine at admission to the ICU |
| NT-pro BNP | Levels of NT-pro Brain natriuretic peptide at admission to the ICU |
| *Outcomes* |  |
| Hypovitaminosis C | Serum level of Vitamin C< 23 μmol/L |
| Vitamin C deficiency | Serum level of Vitamin C<11 μmol/L |
| *Clinical events* |  |
| Mechanical ventilation | 1: use of mechanical ventilation during ICU stay; 0: not using mechanical ventilation |
| AKI stage | Stage 1：Increase in SCr by ≥ 0.3 mg/dL within 48 hours or increase in SCr 1.5 to 1.9 times baseline which is known or presumed to have occurred within the prior 7 days; Stage 2: Increase in SCr to 2.0 to 2.9 times baseline; Stage 3: Increase in SCr to 3.0 times baseline or increase in SCr to ≥ 4.0 mg/dL or initiation of renal replacement therapy. |
| CRRT | 1: subjects need renal replacement therapy, 0: subjects without renal replacement therapy |
| Death | 7-day mortality |

NYHA, new York heart association; NLR, neutrophil lymphocyte ratio; CRP, C-reactive protein; PCT, procalcitonin; Fib, fibrinogen; DD, D-dimer; PT, [prothrombin](javascript:;) [time](javascript:;); LDH, lactic dehydrogenase; Alb, albumin; Cr, creatinine; NT-pro BNP, NT-pro brain natriuretic peptide; AKI, acute kidney injury; RRT, renal replacement therapy.


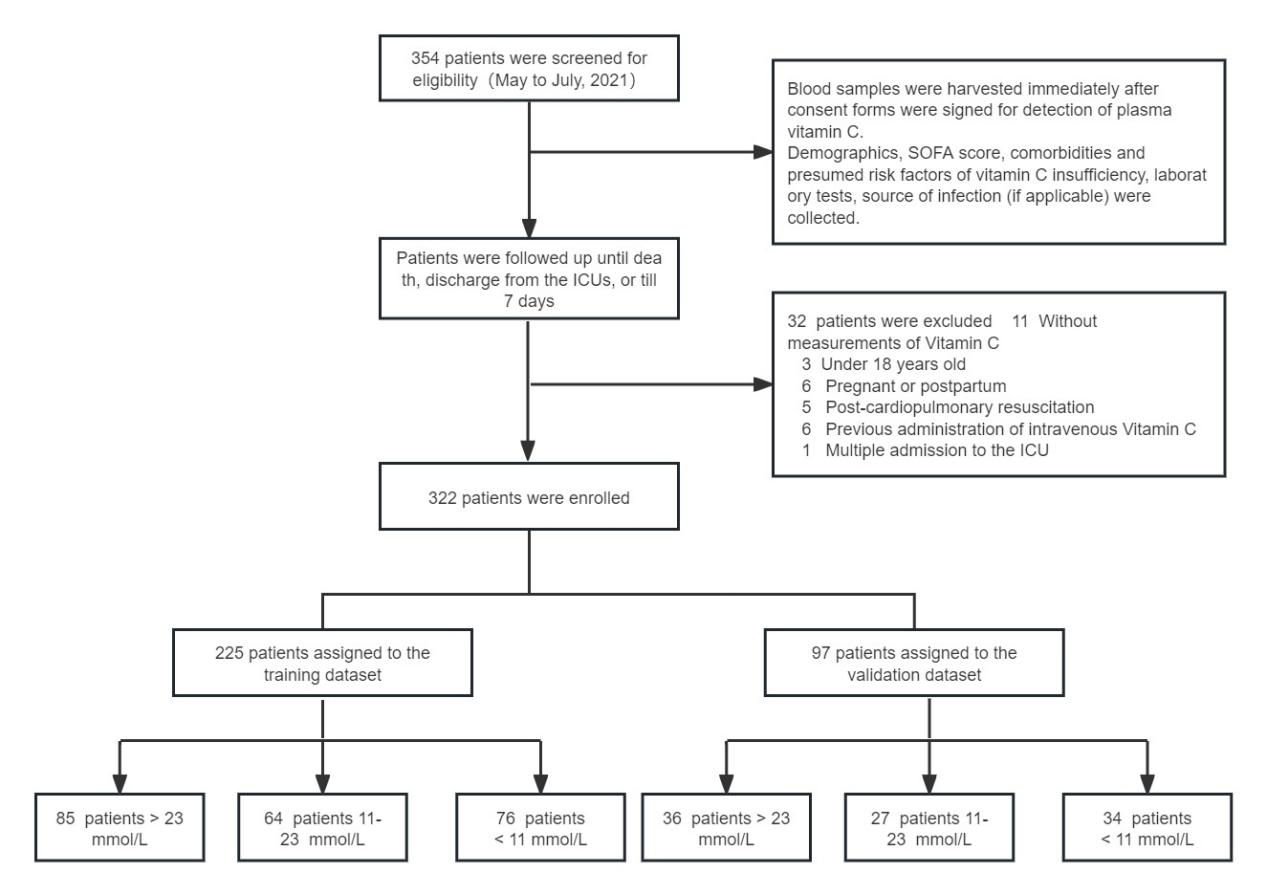


**Fig. S1** Flowchart of the study

**Table S2 Comparison of patient characteristics between the patients with or without hypovitaminosis C**

|  | Overall  (N=322) | Vitamin C ≥ 23  (N=121) | Vitamin C < 23  (N=201) | P value |
| --- | --- | --- | --- | --- |
| Age, yrs, median [IQR] | 68.00  [58.00, 79.00] | 67.00  [55.00, 74.00] | 69.00  [59.00, 80.00] | 0.04 |
| BMI category, n (%) | |  |  | 0.23 |
| 18.5≤BMI<25 | 157 (48.8) | 61 (50.4) | 96 (47.8) |  |
| BMI<18.5 | 33 (10.2) | 7 (5.8) | 26 (12.9) |  |
| 25≤BMI<30 | 111 (34.5) | 44 (36.4) | 67 (33.3) |  |
| BMI≥30 | 21 (6.5) | 9 (7.4) | 12 (6.0) |  |
| Male gender, n (%) | 209 (64.9) | 73 (60.3) | 136 (67.7) | 0.23 |
| Smoker, n (%) | 122 (37.9) | 45 (37.2) | 77 (38.3) | 0.94 |
| Moderate to severe heart failure, n (%) | 22 (6.8) | 6 (5.0) | 16 (8.0) | 0.42 |
| Hypertension, n (%) | 142 (44.1) | 54 (44.6) | 88 (43.8) | 0.97 |
| Diabetes, n (%) | 71 (22.0) | 23 (19.0) | 48 (23.9) | 0.38 |
| Pulmonary disease, n (%) | 29 (9.0) | 12 (9.9) | 17 (8.5) | 0.81 |
| Renal disease, n (%) | 19 (5.9) | 4 (3.3) | 15 (7.5) | 0.20 |
| Digestive disease, n (%) | 28 (8.7) | 13 (10.7) | 15 (7.5) | 0.42 |
| Cancer with chemotherapy, n (%) | 23 (7.1) | 7 (5.8) | 16 (8.0) | 0.61 |
| Abdominal surgery, n (%) | 126 (39.1) | 43 (35.5) | 83 (41.3) | 0.36 |
| Vascular disease, n (%) | 27 (8.4) | 2 (1.7) | 25 (12.4) | 0.002 |
| Sepsis, n (%) |  |  |  | <0.001 |
| Sepsis | 136 (42.2) | 35 (28.9) | 101 (50.2) |  |
| Septic shock | 36 (11.2) | 7 (5.8) | 29 (14.4) |  |
| Source of infection, n (%) | |  |  | <0.001 |
| Abdominal | 80 (24.8) | 17 (14.0) | 63 (31.3) |  |
| Others | 56 (17.4) | 14 (11.6) | 42 (20.9) |  |
| SOFA,  Median [IQR] | 3.00  [2.00, 6.00] | 3.00  [2.00, 4.00] | 4.00  [2.00, 6.00] | <0.001 |
| NLR,  Median [IQR] | 11.65  [6.92, 17.70] | 11.34  [6.14, 15.96] | 11.81  [7.46, 19.21] | 0.09 |
| CRP, mg/dL  Median [IQR] | 2.66  [1.00, 7.14] | 1.45  [0.48, 4.23] | 3.24  [1.57, 8.57] | <0.001 |
| PCT, ng/ml,  Median [IQR] | 0.25  [0.07, 1.29] | 0.14  [0.03, 0.78] | 0.32  [0.12, 1.97] | <0.001 |
| PT, s,  Median [IQR] | 14.50  [13.30, 15.78] | 13.60  [12.50, 14.90] | 14.90  [14.00, 16.60] | <0.001 |
| Fib, g/L,  Median [IQR] | 3.48  [2.76, 4.32] | 3.48  [2.79, 4.32] | 3.47  [2.76, 4.31] | 0.84 |
| DD, μg/ml,  Median [IQR] | 2.72  [1.29, 5.19] | 1.77  [1.04, 4.63] | 3.01  [1.66, 5.37] | 0.001 |
| LDH, U/L,  Median [IQR] | 201.20  [156.25, 282.08] | 195.10  [162.80, 256.00] | 207.50  [155.00, 288.00] | 0.54 |
| Alb, g/L,  Median [IQR] | 32.40  [29.20, 34.98] | 34.20  [30.90, 37.10] | 31.30  [28.30, 33.70] | <0.001 |
| Cr, umol/L,  Median [IQR] | 75.45  [59.70, 103.40] | 72.60  [57.80, 89.40] | 79.00  [61.80, 111.10] | 0.02 |
| NT-pro BNP pg/ml, median [IQR] | 254.90  [85.50, 754.80] | 139.50  [63.00, 380.40] | 320.00  [112.50, 1066.00] | <0.001 |
| Lac, mmol/L,  Median [IQR] | 1.45  [1.00, 2.40] | 1.40  [0.90, 2.50] | 1.50  [1.00, 2.30] | 0.49 |
| MV, n (%) | 243 (75.5) | 85 (70.2) | 158 (78.6) | 0.12 |
| Mechanical ventilation hours, h,  median [IQR] | 18.00  [1.50, 71.75] | 14.00  [0.00, 37.00] | 20.00  [8.50, 90.00] | 0.005 |
| AKI, n (%) | 44 (13.7) | 12 (9.9) | 32 (15.9) | 0.18 |
| CRRT, n (%) | 26 (8.1) | 8 (6.6) | 18 (9.0) | 0.59 |
| Death, n (%) | 13 (4.0) | 1 (0.8) | 12 (6.0) | 0.05 |

**Table S3 Comparison of patient characteristics between the patients with or without Vitamin C deficiency**

|  | level | Overall  (N=322) | Vitamin C ≥ 11  (N=212) | Vitamin C <11  (N=110) | P value |
| --- | --- | --- | --- | --- | --- |
| Age, yrs,  Median [IQR] | | 68.00  [58.00, 79.00] | 67.00  [55.00, 78.00] | 70.00  [61.25, 80.00] | 0.04 |
| BMI, kg/m^2^,  Median [IQR] | | 23.90  [21.07, 26.55] | 24.21  [21.59, 27.06] | 22.86  [20.44, 25.94] | 0.02 |
| BMI category, n (%) | |  |  |  | 0.30 |
| 18.5≤BMI<25 | 1 | 157 (48.8) | 101 (47.6) | 56 (50.9) |  |
| BMI<18.5 | 2 | 33 (10.2) | 18 (8.5) | 15 (13.6) |  |
| 25≤BMI<30 | 3 | 111 (34.5) | 77 (36.3) | 34 (30.9) |  |
| BMI≥30 | 4 | 21 (6.5) | 16 (7.5) | 5 (4.5) |  |
| Male gender, n (%) | | 209 (64.9) | 135 (63.7) | 74 (67.3) | 0.61 |
| smoker, n (%) | | 122 (37.9) | 80 (37.7) | 42 (38.2) | 1.00 |
| Moderate to severe heart failure, n (%) | | 22 (6.8) | 202 (95.3) | 98 (89.1) | 0.06 |
| Hypertension, n (%) | | 142 (44.1) | 96 (45.3) | 46 (41.8) | 0.63 |
| Diabetes, n (%) | | 71 (22.0) | 43 (20.3) | 28 (25.5) | 0.36 |
| Pulmonary disease, n (%) | | 29 (9.0) | 17 (8.0) | 12 (10.9) | 0.51 |
| Wasting of Vitamin C by the kidney, n (%) | | 19 (5.9) | 10 (4.7) | 9 (8.2) | 0.32 |
| Digestive disease, n (%) | | 28 (8.7) | 21 (9.9) | 7 (6.4) | 0.39 |
| Cancer with chemotherapy, n (%) | | 23 (7.1) | 16 (7.5) | 7 (6.4) | 0.87 |
| Abdominal surgery, n (%) | | 126 (39.1) | 81 (38.2) | 45 (40.9) | 0.73 |
| Vascular disease, n (%) | | 27 (8.4) | 10 (4.7) | 17 (15.5) | 0.002 |
| Sepsis, n (%) |  |  |  |  | <0.001 |
| Sepsis | 1 | 136 (42.2) | 80 (37.7) | 56 (50.9) |  |
| Septic shock | 2 | 36 (11.2) | 16 (7.5) | 20 (18.2) |  |
| Source of infection, n (%) | | |  |  | <0.001 |
| Abdominal | 1 | 80 (24.8) | 44 (20.8) | 36 (32.7) |  |
| Others | 2 | 56 (17.4) | 29 (13.7) | 27 (24.5) |  |
| SOFA score,  median [IQR] | | 3.00  [2.00, 6.00] | 3.00  [2.00, 5.00] | 5.00  [2.00, 7.00] | 0.001 |
| NLR, median [IQR] | | 11.65  [6.92, 17.70] | 11.16  [6.45, 17.30] | 12.15  [8.07, 19.52] | 0.10 |
| CRP, mg/dL,  median [IQR] | | 2.66  [1.00, 7.14] | 1.92  [0.75, 5.15] | 4.75  [2.13, 8.84] | <0.001 |
| PCT, ng/dL,  median [IQR] | | 0.25  [0.07, 1.29] | 0.17  [0.05, 1.06] | 0.55  [0.17, 1.97] | <0.001 |
| PT, s,  median [IQR] | | 14.50  [13.30, 15.78] | 14.20  [12.90, 15.43] | 14.90 [14.10, 16.70] | <0.001 |
| Fib, g/L,  median [IQR] | | 3.48  [2.76, 4.32] | 3.45  [2.72, 4.32] | 3.55  [2.84, 4.30] | 0.77 |
| DD, μg/ml,  median [IQR] | | 2.72  [1.29, 5.19] | 2.62  [1.19, 5.20] | 2.95  [1.88, 5.13] | 0.07 |
| LDH, U/L,  median [IQR] | | 201.20  [156.25, 282.08] | 195.05  [156.75, 265.50] | 227.70  [156.45, 292.82] | 0.20 |
| Alb, g/L,  median [IQR] | | 32.40  [29.20, 34.98] | 33.00  [29.85, 35.50] | 31.50  [28.25, 33.50] | 0.002 |
| Cr, umol/L,  median [IQR] | | 75.45  [59.70, 103.40] | 73.70  [59.15, 93.73] | 80.85  [61.32, 116.12] | 0.07 |
| NT-pro BNP, pg/dL, median [IQR] | | 254.90  [85.50, 754.80] | 185.40 [69.75, 538.30] | 396.65  [173.18, 1227.50] | <0.001 |
| Lac, mmol/L,  median [IQR] | | 1.45  [1.00, 2.40] | 1.40  [1.00, 2.50] | 1.50  [1.00, 2.20] | 0.85 |
| MV, n (%) | | 243 (75.5) | 153 (72.2) | 90 (81.8) | 0.08 |
| Mechanical ventilation hours, h, median [IQR] | | 18.00  [1.50, 71.75] | 15.00  [0.00, 47.25] | 21.00  [12.25, 90.00] | 0.004 |
| AKI, n (%) | | 44 (13.7) | 23 (10.8) | 21 (19.1) | 0.06 |
| RRT, n (%) | | 26 ( 8.1) | 13 (6.1) | 13 (11.8) | 0.12 |
| Death, n (%) | | 13 (4.0) | 4 (1.9) | 9 (8.2) | 0.02 |

**Fig. S2** The prediction model for hypovitaminosis C established by LASSO regression with five fold cross-validation.

**Fig. S3** Evaluation and validation of prediction model for Vitamin C Deficiency.

The ROC curve, Calibration curve, Decision curve analyses for nomogram in the training set(a, c, e) and testing set (b, d, f).


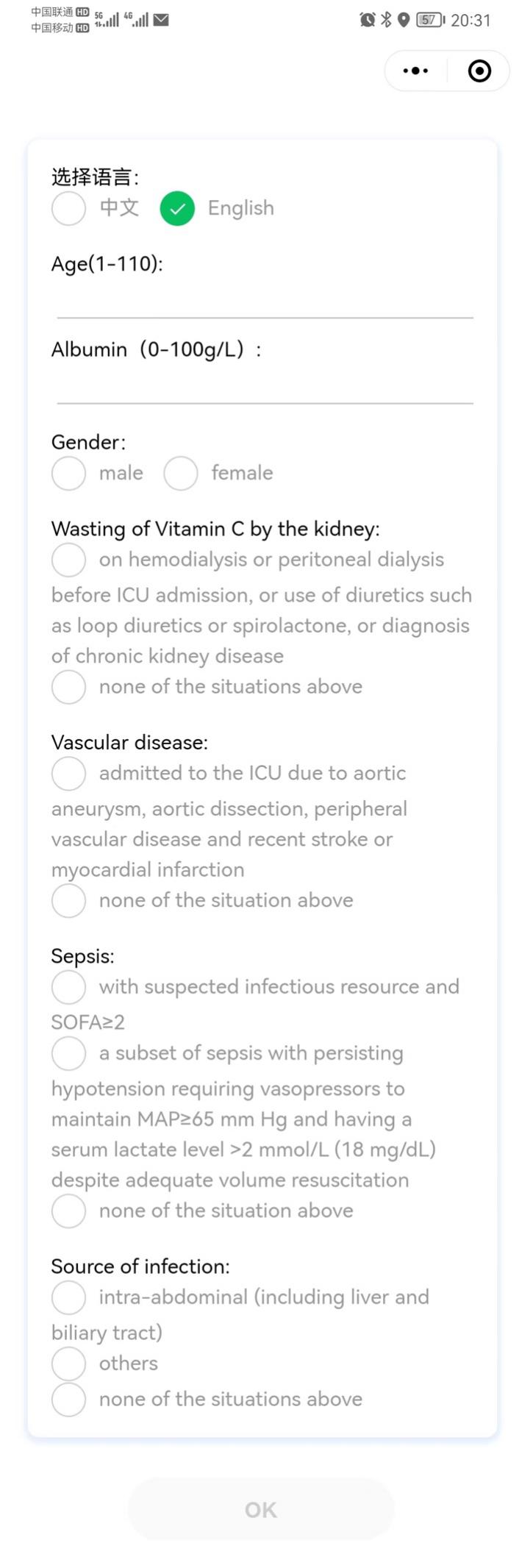


**Fig. S4** Screenshot of WeChat App.

**Table S4 Odds ratio for Vitamin C deficiency in univariable and multivariable** **analyses**

| Characteristics | Univariable analysis | | | Multivariable analysis | | |
| --- | --- | --- | --- | --- | --- | --- |
|  | **OR** | **95%CI** | **P value** | **OR** | **95%CI** | **P value** |
| Age | 1.02 | 1.00~1.04 | 0.01 | 1.02 | 1.00~1.04 | 0.02 |
| Male gender | 1.17 | 0.72~1.91 | 0.52 |  |  |  |
| Body Mass Index |  |  |  |  |  |  |
| BMI<18.5 | 0.56 | 0.20~1.62 | 0.29 |  |  |  |
| 25≤BMI<30 | 0.80 | 0.47~1.34 | 0.39 |  |  |  |
| BMI≥30 | 1.50 | 0.70~3.21 | 0.29 |  |  |  |
| Smoker | 1.02 | 0.63~1.64 | 0.94 |  |  |  |
| Moderate to severe heart failure | 2.47 | 1.03~5.92 | 0.04 |  |  |  |
| Hypertension | 0.87 | 0.55~1.38 | 0.55 |  |  |  |
| Diabetes | 1.34 | 0.78~2.31 | 0.29 |  |  |  |
| Pulmonary disease | 1.40 | 0.65~3.06 | 0.39 |  |  |  |
| Wasting of Vitamin C by the kidney | 1.80 | 0.71~4.57 | 0.22 | 1.87 | 0.68~5.12 | 0.23 |
| Digestive system disorder | 0.62 | 0.25~1.50 | 0.29 |  |  |  |
| Cancer with chemotherapy | 0.83 | 0.33~2.09 | 0.70 |  |  |  |
| SOFA score | 1.10 | 1.03~1.17 | 0.006 |  |  |  |
| Sepsis |  |  |  |  |  |  |
| Sepsis | 2.39 | 1.43~3.99 | 0.001 | 1.55 | 0.75~3.22 | 0.24 |
| Septic shock | 4.26 | 1.99~9.12 | 0.000 | 2.68 | 1.01~7.15 | 0.04 |
| Source of infection |  |  |  |  |  |  |
| Abdominal | 2.42 | 1.39~4.20 | 0.002 | 1.76 | 0.82~3.79 | 0.15 |
| Others | 2.75 | 1.48~5.12 | 0.001 | 2.14 | 0.93~4.90 | 0.07 |
| Abdominal surgery | 1.12 | 0.70~1.79 | 0.64 |  |  |  |
| Vascular disease | 3.69 | 1.63~8.37 | 0.002 | 4.50 | 1.84~11.02 | 0.001 |
| NLR | 1.00 | 0.98~1.01 | 0.93 |  |  |  |
| CRP | 1.06 | 1.02~1.11 | 0.004 |  |  |  |
| PCT | 1.00 | 0.98~1.01 | 0.79 |  |  |  |
| Fib | 0.96 | 0.81~1.14 | 0.67 |  |  |  |
| PT | 1.07 | 1.00~1.14 | 0.04 |  |  |  |
| DD | 1.01 | 0.96~1.07 | 0.66 |  |  |  |
| LDH | 1.00 | 1.00~1.00 | 0.95 |  |  |  |
| Alb | 0.92 | 0.88~0.97 | 0.001 |  |  |  |
| Cr | 1.00 | 1.00~1.01 | 0.03 |  |  |  |
| NT-pro BNP | 1.00 | 1.00~1.00 | 0.59 |  |  |  |
| Lac | 1.01 | 0.91~1.13 | 0.79 |  |  |  |

**Fig. S5** The prediction model for vitamin C deficiency established by LASSO regression with five fold cross-validation.

**Table S5 Predictive performance of the model generated to Vitamin C deficiency**

|  | AUC | ACC | Sensitivity | Specificity | F1 | PPV | NPV |
| --- | --- | --- | --- | --- | --- | --- | --- |
| Performance | 0.72  [0.66-0.77] | 0.69  [0.64-0.73] | 0.16  [0.10-0.23] | 0.96  [0.93-0.98] | 0.26  [0.16-0.36] | 0.67  [0.46-0.84] | 0.69  [0.64-0.74] |

*AUC* area under the curve of receiver operating characteristics, *PPV* positive predictive value, *NPV* negative predictive value.

**Fig. S6** The performance of prediction model for vitamin C deficiency.
